# Hypertension in the Elderly

**DOI:** 10.1155/2012/859176

**Published:** 2011-08-16

**Authors:** Blas Gil-Extremera, Pedro Cía-Gómez

**Affiliations:** ^1^Service of Internal Medicine, Hypertension and Lipid Unit, “San Cecilio” University Hospital, Granada, Spain; ^2^Department of Medicine, The University of Zaragoza, Zaragoza, Spain

## Abstract

*Background*. The incidence of hypertension in the Western countries is continuously increasing in the elderly population and remains the leading cause of cardiovascular and morbidity. *Methods*. we analysed some significant clinical trials in order to present the relevant findings on those hypertensive population. *Results*. Several studies (SYST-EUR, HYVET, CONVINCE, VALUE, etc.) have demonstrated the benefits of treatment (nitrendipine, hydrochrotiazyde, perindopril, indapamide, verapamil, or valsartan) in aged hypertensive patients not only concerning blood pressure values but also the other important risk factors. *Conclusion*. Hypertension is the most prevalent cardiovascular disorder in the Western countries, and the relevance of receiving pharmacological treatment of hypertension in aged patients is crucial; in addition, the results suggest that combination therapy—nitrendipine plus enalapril—could have more benefits than those observed with the use of nitrendipine alone.

## 1. Introduction

Hypertension remains the first cause of cardiovascular risk and mortality worldwide, and, in people aged 65 or older, it has been duplicated in relation to four decades before. Also population aged 80 or older is increasing; the prevalence of hypertension in this group of people is above 60% and continues to grow. It is well known that hypertension is *per se* associated to other risk factors such as overweight, obesity (46.8%), tobacco abuse, or hypercholesterolemia (45.4%) [1]. It is estimated that during the next years, 75% of the medical practice will be aimed at the geriatric age group. Today, everybody wishes to reach elderly without ailments or clinical disorders that could seriously alter his quality of life. Hypertension is one of the main medical problems affecting the elderly. Pharmacological therapeutics is especially aimed at studying that very common medical condition. During the last years, and before the final findings of the Syst-Eur were published [2], the general medical opinion considered not to decrease blood pressure values in order to avoid possible ischemic events and poor oxygenation of the trigger organs (brain, myocardium, kidney, and visual organ). This strong belief within the medical community raised the question of whether or not aged people should receive pharmacological treatment similarly to other younger patients. This scientific question was raised at the beginning of the 90s about hypertensive people aged 60–79. Until then, many clinicians thought that it was harmful to normalize blood pressure values of aged hypertensive people because of the hypothetical danger of ischemic events in the trigger organs and, then, the possibility to provoke serious cardiovascular complications.

## 2. Methods

However, the results of the *Systolic Hypertension in Europe trial *(*Syst-Eur*), where our group has actively participated, clearly proved the benefits of hypertensive treatment versus placebo, in contrast to the nihilistic attitude to leave the disease to itself. The significant results of the Syst-Eur trial are based on the research and the ten-year followup of 4,227 patients from 23 European countries. The protocol of this trial has been described in detail before [2]. These findings demonstrated (1) a reduction in cardiac mortality in the group of patients treated with antihypertensive agents (nitrendipine, enalapril, and hydrochlorothiazide); (2) a lower incidence of stroke, angina pectoris, and myocardial infarction in the group receiving active treatment; (3) a decrease in the incidence of dementia in this group. The potential reduction up to 50% of dementia episodes after treatment with antihypertensive agents was based on the use of nitrendipine (dihydropyridine calcium antagonist) as the first drug with an important role in increasing the longevity of the elderly all over the world; these findings also showed that the nonpharmacological intervention in this disease must not be neglected [3, 4]. The results of the Syst-Eur trial suggest that combination therapy of nitrendipine plus enalapril could be interesting in a better prognosis of hypertensive patients [5]. In conclusion, the benefits of the antihypertensive treatment in patients with hypertension aged 60 or older revealed a reduction in the cases of stroke and cardiovascular events, which has been confirmed by other studies and meta-analysis of the last years.

## 3. Results

Also, it is well-known that life expectancy is rising around the world which is related to an increased risk of developing dementia; this is becoming a major public health problem. The Syst-Eur study showed that randomized patients actively treated presented a lower incidence of dementia up to 50% (from 7.7 to 3.8 cases every 1000 patients/yr) (21 cases versus 11 cases; *P* = 0.05) compared to the group treated with placebo.

Based on the placebo group, we can extrapolate that five-year treatment of 1,000 patients with isolated systolic hypertension could prevent 19 cases of dementia [6]. Therefore, the conclusive results of the Syst-Eur trial showed that physicians (internists, cardiologists, primary care, endocrinologists, and others) should treat hypertension drastically in aged patients and try to reach blood pressure values similar to those recommended in younger populations (≤130/80 mmHg); these values should be lower when patients have also diabetes (≤125/75 mmHg).

On the other hand, several observations suggested that calcium antagonists could have a specific neuroprotective effect. It is well known that both in vascular and mixed or degenerative dementias the values of the minimental state examination (MMSE) are less decreased in patients treated with nimodipine than in those treated with placebo. Brain aging is accompanied by alterations of intracellular calcium regulation, which leads to several cellular disorders, resulting in cellular death. Disorders of calcium homeostasis are related to the brain's aging process and the Alzheimer's disease. Antihypertensive treatment and reduction in blood pressure levels decrease the risk of developing dementia in aged patients, which is an important finding for the public healthcare system; calcium antagonists (nitrendipine and nimodipine) can have a specific neuroprotective effect. Important advances have been achieved relating physiopathological, pathogenetic, and therapeutic mechanisms of arterial hypertension. However, few studies have been made until now including the treatment of hypertension in patients aged 80 or older. These significant results led the researchers to investigate whether the benefits of the Syst-Eur trial could be extrapolated to patients aged 80 or older. However, empiric therapy is not adequate to this important pathology. The question was the *primum movens* of the HYVET study (Hypertension in the Very Elderly Trial), which was designed to provide a clear, definitive, and scientific answer to the treatment of hypertension in individuals older than 80 years.

The adequate control of these patients is greatly important for the general practitioners, internists, geriatrists, and other specialists, since hypertension together with other chronic conditions such as diabetes, hypercholesterolemia, and obesity are the main pathogenetic factors of cardiovascular disease, which is today the first cause of morbidity and mortality in the Western countries. The risks and benefits of treating hypertensive patients were studied in the HYVET trial, in which our group participated actively. The pilot study was carried out in 1,283 patients and was supported by the British Heart Foundation. Three different therapies were designed: (1) no treatment; (2) low-dose diuretic-based regimen (hydrochlorothiazide 12.5–25 mg daily); (3) an angiotensin-converting enzyme inhibitor (lisinopril 5 mg or enalapril 10 mg), following accurate inclusion and follow-up clinical criteria, and quality of results and procedures carried out according to protocol. The cases studied (1,283 patients) were recruited from several European countries (Bulgaria, Spain, Romania, UK, Poland, Finland, Lithuania, Ireland, Greece, and Serbia) [7, 8]. This study demonstrated that treating 1,000 patients per year, 19 cases of stroke could be prevented (nine of them nonfatal stroke). The good results of the study led to continue the investigation (“Main HYVET Trial”) as international randomized double-blind trial. 

Around 5,000 patients were included in the study. In conclusion, HYVET trial demonstrated the benefits of reducing blood pressure values in patients aged 80 or older and provided clear evidences about the need to treat hypertension in this growing population; this has become very important in the clinical practice and therapeutic guides [9, 10]. For many years, physicians have had the mistaken idea that hypertension was a result of ageing and that treatment was not necessary and no common criteria existed about the therapy on these cases; some proposed no treatment, others suggested low-dose diuretics, and others recommended the use of other pharmacological groups (angiotensin-converting enzymes inhibitors, calcium channel blocker, or angiotensin-II receptor antagonists). It is well established that hypertension is frequently associated with several cardiovascular risk factors, such as obesity, hypercholesterolemia, diabetes, tobacco abuse, and others. This fact led to carry out new clinical trials including hypertensive population with other cardiovascular events, like CONVINCE (Controlled Onset Verapamil Investigation of Cardiovascular Endpoints) study; this is a double-blind, randomized, multicentre, international trial which compared two initial treatments [11]. 

At the beginning, physicians could choice the treatment with hydrochlorothiazide (HCTZ) or atenolol, and the possibility to add controlled-onset extended-release verapamil, if necessary. The protocol permitted an increase of the doses or the number of drugs until reaching values lower than 140/90 mmHg. A total of 16,602 patients were included in the study selected from 15 countries and 661 centres. The mean age of the patients was 65.6 ± 7.4 years. The main risk factors of the participants were overweight (BMI ≥ 28.5/m^2^, 50.4%); dyslipemia, 31.3%; tobacco abuse 22.6%; diabetes, 19.8%, and in a lower incidence others such as known vascular disease, left ventricular hypertrophy, previous myocardial infarction, transient cerebral ischaemia, and so forth. Results showed a similar efficiency in the reduction of the cardiovascular disease with the calcium antagonist agents than with diuretics and beta blockers agents [12].

There have been many therapeutic advances since the development of reserpine and phenobarbital as therapeutic agents, more than five decades ago. However, despite the excellent advances of the last years, hypertension continues to be the main factor of complication and cardiac death not only in the Western population, mostly present as cerebral infarction and coronary heart disease. At the moment, the number of patients with controlled hypertension is under 25% all over the world and this could be due to the following reasons: (1) a difficult access to the healthcare system, (2) the lack of symptoms of hypertension, (3) the side effects of the antihypertensive agents, (4) the presence of other risk factors (obesity, tobacco abuse, diabetes, hypercholesterolemia, etc.), (5) the concomitance of other associated noncardiovascular diseases (pneumopathy, digestive disorders, and osteoarticular disorders), and (6) the difficulty in adjusting dosage.

Despite the therapeutic advances, uncontrolled hypertension is a growing problem in the developed countries and populations having easy access to the healthcare system. Unfortunately, not all the hypertensive patients receive treatment when the values of the diastolic blood pressure are 90–95 mmHg, specially, when only systolic blood pressure values between 140–160 mmHg are considered. Therefore, the aim to reach values ≤130 mmHg is still far away. This is a very important problem, since in hypertensive people brain alterations secondary to vascular diseases lead to intellectual impairment and a worse quality of life of these patients [13, 14]. 

The ACTION study [15] was carried out in 7,665 patients (mean age 63.5 years) randomly assigned to treatment with nifedipine GITS (gastrointestinal therapeutic system) and 3,840 patients treated with placebo. The study demonstrated that after addition of nifedipine GITS to the conventional treatment of angina pectoris, no effect was observed on major cardiovascular event-free survival, and it reduced the need to perform angiography and coronary interventions.  Table 1 shows some findings (number of patients, age, and drugs used) on clinical trials in aged hypertensive people.

## 4. Conclusion

Hypertension is a very important disorder in aged people and is associated with higher risk of cardiovascular morbidity and mortality. The fact of reducing blood pressure values decreases the risk for cardiac death as well as neurological, metabolic, and musculoskeletal system sequelae in aged people. Therefore, the aim of the antihypertensive treatment must be to reduce cardiovascular risks and to maintain an adequate quality of life and good functional capacity in these patients.

## Figures and Tables

**Table 1:** main clinical trials on hypertensive patients.

Acronym	Patients	Age	Drugs
ACTION	7.665	63.5*	Nifedipine GITS
ALLHAT	40.000	≥55 years	Chortalidone, amlodipine, lisinopril, doxazosin, pravastatin
ELITE	722	≥65	Losartan, captopril
HOT	18.790	50–80	Felodipine, hydroclorotiazide, atenolol, ACEI
INSIGH	6.321	55–80	Nifedipine, amiloride
SYST-EUR	4.227	60–79	Nitrendipine, enalapril, hydroclorotiazide
ANBP_2_	6.000	65–84	Enalapril versus *ββ*/*αβ*/diuretic/antiCa^++^
CAPP	7.000	25–66	Captopril versus *ββ*/diuretics
LIFE	9.193	55–85	Losartan versus atenolol
NORDIL	10.881	50–69	Diltiazem versus *ββ*/diuretic
PROGRESS	6.000	64*	Perindopril. Indapamide
CONVINCE	16.602	>55	Verapamil** versus/atenolol/hydroclorotiazide
VALUE	14.400	>50	Valsartan, amlodipine
HYVET	2.100	>80	Indapamide, perindopril

*Median Age;  **Verapamil Slow Release.
